# Hematological Disorders following Gastric Bypass Surgery: Emerging Concepts of the Interplay between Nutritional Deficiency and Inflammation

**DOI:** 10.1155/2013/205467

**Published:** 2013-07-25

**Authors:** Mingyi Chen, Amrita Krishnamurthy, Ali R. Mohamed, Ralph Green

**Affiliations:** ^1^Department of Pathology and Laboratory Medicine, University of California Davis Medical Center, Sacramento, CA 95817, USA; ^2^Department of Surgery, University of California Davis Medical Center, Sacramento, CA 95817, USA

## Abstract

Obesity and the associated metabolic syndrome are among the most common and detrimental metabolic diseases of the modern era, affecting over 50% of the adult population in the United States. Surgeries designed to promote weight loss, known as bariatric surgery, typically involve a gastric bypass procedure and have shown high success rates for treating morbid obesity. However, following gastric bypass surgery, many patients develop chronic anemia, most commonly due to iron deficiency. Deficiencies of vitamins B1, B12, folate, A, K, D, and E and copper have also been reported after surgery. Copper deficiency can cause hematological abnormalities with or without neurological complications. Despite oral supplementation and normal serum concentrations of iron, copper, folate, and vitamin B12, some patients present with persistent anemia after surgery. The evaluation of hematologic disorders after gastric bypass surgery must take into account issues unique to the postsurgery setting that influence the development of anemia and other cytopenias. In this paper, the clinical characteristics and differential diagnosis of the hematological disorders associated with gastric bypass surgery are reviewed, and the underlying molecular mechanisms are discussed.

## 1. Introduction

The worldwide pandemic of obesity carries alarming health and socioeconomic implications. Obesity is a significant risk factor for the onset of metabolic disorders, including type 2 diabetes, hyperlipidemia, cardiovascular disease, stroke, and many other chronic diseases. More than 300 million people worldwide are obese [1]. In the United States, more than one-third of the population is overweight, and approximately 15 million people (about 5%) are considered morbidly obese (defined as a body mass index greater than 40) [1]. Bariatric surgery has proven to be the most effective treatment for morbid obesity [2]. In 2008, about 220,000 bariatric and metabolic operations were performed in the United States. Despite a major evolution in bariatric surgery over the past two decades, the Roux-en-Y gastric bypass procedure (RYGB) remains the most effective bariatric operation in the United States [2]. This popular procedure entails the long-term complications of hematological disorders, especially chronic anemia [3].

The spectrum of hematologic disorders after gastric bypass surgery includes single or multilineage cytopenias resulting from a complex interrelationship of micronutritional deficiencies and inflammatory, immune-mediated, infectious, or drug-related factors [3]. This condition contributes to diminished quality of life and reduced physical and cognitive function. The evaluation of hematologic disorders after gastric bypass surgery must take into account issues unique to the postsurgery setting. This review will focus on the factors unique to RYGB that influence the development of anemia and other cytopenias. Although micronutrient deficiencies play a significant role, recent studies suggest a possible role for adipocyte-derived circulating inflammatory cytokines and hormones in the development of hematopoietic abnormalities [4]. Further studies need to be performed to elucidate the interactions that contribute to the development of hematological abnormalities following gastric bypass surgery.

## 2. Bariatric Surgery

**Figure 1:**
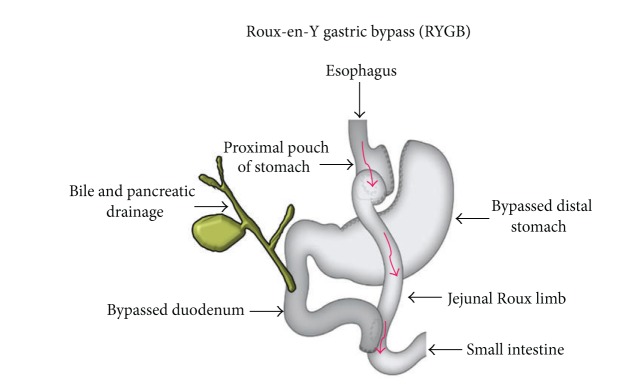
Diagrammatic representation of gastric bypass using a Roux-en-Y anastomosis.

Bariatric surgery is the term encompassing all of the surgical treatments for obesity. These operations are prescribed to treat morbid obesity, type 2 diabetes, hypertension, sleep apnea, and other comorbid conditions. Bariatric surgery is the most effective current treatment for morbid obesity in order to produce significant and sustained weight loss as well as markedly reduced comorbidities [2]. There are two main principles of bariatric surgery which exist either in combination or alone: restriction and malabsorption. The common bariatric procedures include laparoscopic vertical-banded gastroplasty, gastric binding, and Roux-en-Y gastric bypass. Among the bariatric procedures, the “divided” or “disconnected” purely restrictive operations limit the amount of solid food that can be consumed. The most common procedure worldwide is the Roux-en-Y gastric bypass (RYGB) which is both a restrictive and malabsorptive operation (Figure 1). RYGB is currently the most commonly performed bariatric procedure in USA and Canada while adjustable gastric banding is the most popular bariatric procedure in Europe.

## 3. Gastric Bypass Procedure: The Roux-en-Y Gastric Bypass

The gastric bypass procedure (GBP), in its various forms, accounts for a large majority of the bariatric surgical procedures performed. Gastric bypass first divides the stomach into a small upper pouch and a larger lower “remnant” pouch; the small intestine is then rearranged and connected to both pouches. Surgeons have developed several different ways to reconnect the intestine, thus leading to several different GBP variants. Any GBP leads to a marked reduction in the functional volume of the stomach, accompanied by an altered physiological and physical response to food.

According to the American Society for Bariatric Surgery and the National Institutes of Health, the number 1 weight loss surgery performed in the USA is the Roux-en-Y gastric bypass [5]. The surgery is usually performed by noninvasive laparoscopic surgery and is considered the gold standard bariatric surgery procedure. 

This procedure involves the formation of a small (<30 mL) stomach pouch that drains food into an alimentary or Roux limb (Figure 1), while the remainder of the stomach secretes gastric acid, pepsin, and intrinsic factor into a biliopancreatic limb which combines with pancreatic enzymes and bile and joins the alimentary (Roux) limb approximately 100 cm from the gastrojejunostomy. This procedure shortens the small intestine but still allows for digestion and absorption. Roux-en-Y bypass reducing the appetite of patient is supposed to be associated with increased satiety gut hormone concentrations [2]. 

## 4. Benefits of Gastric Bypass Procedure

Obesity is associated with multiple comorbidities, including hypertension, hypercholesterolemia, hypertriglyceridemia, diabetes mellitus, obstructive sleep apnea, osteoarthritis, back/extremity pain, gastroesophageal reflux disease (GERD), asthma, and depression. A quick and dramatic weight loss has been seen within the first year postoperatively. Sustained weight loss has been reported up to 16 years postoperatively with gastric bypass [2]. Gastric bypass surgery can significantly improve or resolve many of the debilitating obesity-related comorbidities to reduce long-term mortality [6].

Patients who undergo Roux-en-Y surgery usually lose about 65 to 80% of their excess body weight in the first year after surgery [2]. A dramatic reduction of the following health conditions is also achieved. Hyperlipidemia (high cholesterol and high triglyceride levels) is corrected in over 70% of patients. Essential hypertension is reduced in over 70% of patients, and medication requirements are usually reduced in the remainder. Sleep apnea improves substantially in most patients. Diabetes type 2 is reversed in up to 90% of patients usually leading to a normal blood sugar without medication, sometimes within days of surgery. Patients are able to enjoy greater social participation [2].

## 5. Pathophysiology of Gastric Bypass Surgery

Gastric bypass surgery (RYGB) alters both the anatomy and physiology of the stomach and small intestine. It reduces the size of the stomach by well over 90%. A normal stomach can stretch, sometimes to over 1000 mL, while the pouch of the gastric bypass is limited to 15–30 mL in size. It is still generally perceived that the profound weight loss effects of gastric bypass are ascribed to mechanical food restriction and/or malabsorption. However, recent clinical and animal studies suggest that the increase in resting energy expenditure plays a significant role [3]. Thus, it appears that RYGB affects weight loss by altering the physiology of weight regulation and eating behavior rather than by simple mechanical restriction or malabsorption alone [2].

Nutritional deficiencies are very common after RYGB and occur despite supplementation with the standard multivitamin preparation [7]. The deficiencies appear to be more substantial following malabsorptive procedures such as gastric bypass procedures, but occur with restrictive procedures as well. Anemia is common following RYGB, and multiple mechanisms contribute to its development in different stages of the clinical setting [8]. Immediately after surgery, anemia due to inappropriately low levels of erythropoietin or iron deficiency is common. In addition, suppression of erythropoiesis and interference with the production of erythropoietin or its signaling pathway may contribute to decreased production of red blood cells. In the long term, the estimated incidence of anemia following RYGB surgery varies from 12% to 30%, mostly ascribed to micronutrient including vitamin-deficiency states [9]. Multiple micronutrient deficiencies occur, including the major hematinic factors, iron and vitamin B12. Deficiencies of vitamins B1, A, K, D, and E have also been reported after surgery [10]. In addition to iron and vitamin B12 deficiency, copper deficiency has also been reported after RYGB, which can cause hematological abnormalities with or without associated neurological complications [11]. Yet, despite oral supplementation and normal blood concentrations of iron, copper, folic acid, and vitamin B12, many patients display persistent anemia after RYGB. These hematological complications are of clinical significance since anemia often has serious comorbid consequences.

Adipose tissue has been shown to have a role in mediating biological effects on metabolism and inflammation, including the maintenance of energy homeostasis and the pathogenesis of obesity-related metabolic complications. Obesity is associated with chronic low-grade systemic inflammation [4, 12]. There is growing evidence that the obesity induced chronic inflammatory milieu plays a causal role in the development of insulin resistance, type 2 diabetes, and metabolic syndrome. Adipocytes are now recognized to be major secretory cells producing fatty acids as well as over 50 cytokines, collectively referred to as adipokines. A number of adipokines are linked to the inflammatory response, including adiponectin, C-reactive protein (CRP), tumor necrosis factor-*α* (TNF-*α*), interleukin-1*β* (IL-1*β*), IL-6, IL-8, IL-10, monocyte chemoattractant protein-1, macrophage migration inhibitory factor, nerve growth factor, vascular endothelial growth factor, plasminogen activator inhibitor-1, and haptoglobin [13]. Obesity also increases hepcidin levels and is associated with diminished response to oral iron therapy in patients with iron deficiency anemia [14, 15]. Although iron deficiency is a prominent cause for anemia in obese patients after bariatric surgery, it does not account for all cases. The excess iron supplementation and overload with blood transfusions can cause secondary hemosiderosis and irreversible liver injury. Therefore, bariatric surgery patients require appropriate postsurgical management which includes lifelong followup of hematological and metabolic parameters [7]. 

## 6. Evaluation of Anemia and Cytopenias after RYGB

RYGB is also associated with a generalized decrease of white blood cell and platelet counts accompanied by significant anemia. These decreases of WBC and platelet counts do not appear to be clinically significant, unlike the substantial decrease in red blood cell mass. A generalized suppression of hematopoiesis might occur after RYGB and explain some of the phenomena [16]. Hemolysis and a reduced erythrocyte survival may also be of importance as a cause of anemia immediately after bariatric surgery [3].

### 6.1. Iron Deficiency

Iron deficiency after RYGB can manifest as anemia which occurs in 6–50% of patients who undergo gastric bypass surgery [3]. Iron malabsorption is related primarily to disruption of the absorption of iron which normally takes place from the duodenum and proximal jejunum, and which are bypassed at the time of RYGB [17]. In addition, bacterial overgrowth and the induction of an iron-losing enteropathy may play a role. Notably, infection with *Helicobacter pylori *is known to alter gastric pH in combination with hypergastrinemia such that iron absorption is impaired [18]. It is not clear how important the absence of acid in the gastric pouch might be for enhancing the solubility of nonheme food iron [3].

Iron deficiency is usually characterized by microcytic hypochromic anemia. The CBC typically shows low hemoglobin (HGB), hematocrit (HCT), mean corpuscular volume (MCV), mean corpuscular hemoglobin (MCH) and/or mean corpuscular hemoglobin concentration (MCHC), and high red blood cell distribution width (RDW) [19]. Iron storage depletion is detected by diagnostic tests such as low serum ferritin, and iron restricted erythropoiesis is indicated by lowered serum iron level, elevated serum transferrin, and total iron binding capacity (TIBC). A low serum ferritin is the most specific laboratory test for iron deficiency anemia. However, serum ferritin, an acute phase reactant and marker of inflammation, can be elevated by any type of inflammation and so is not always a reliable test of iron status if it is within normal limits (i.e., this test is meaningful if it is abnormally low, but less meaningful if it is normal) [19]. The transferrin saturation index can also be used as an indicator of tissue iron deficiency. A saturation <5% generally indicates iron deficiency but may also occur in association with inflammation, although the levels are usually not as low as those encountered in iron deficiency. Levels from 5% to 10% make the diagnosis of iron deficiency possible, but not definitive. Saturations over 12% may also be associated with iron deficiency, particularly in the setting of inflammation. Transferrin saturation is associated with both transferrin concentrations and serum iron levels, which vary diurnally, and thus should be examined along with serum ferritin in order to assess iron status [2, 8, 20].

Obesity-induced chronic inflammation leads to activation of the immune system that causes alterations of iron homeostasis, mediated through hepcidin, and leads to hypoferremia, iron-restricted erythropoiesis, and finally mild-to-moderate anemia [8, 15]. Obesity increases hepcidin levels and is associated with diminished response to oral iron therapy in patients with iron deficiency anemia [21]. Although iron deficiency is a prominent cause of anemia in patients after RYGB, it does not account for all cases. Perturbation of iron utilization, as seen in the anemia associated with inflammation, also plays an important role. Therefore, anemia after bariatric surgery cannot simply be explained on the basis of iron availability which suggests that other mechanisms, currently undefined, contribute to the development of anemia in these patients. RYGB patients require lifelong followup of hematological and metabolic parameters as well as appropriate management tailored to the need of individual patients. Both the innate and adaptive immune systems are worthy of further interrogation of their role in the regulation of chronic inflammation and metabolism in refractory anemia following RYGB.

If the anemia is persistent and unresponsive to iron supplementation therapy, additional micronutrient deficiencies and other origins of the anemia, such as *H. pylori *infection, must be considered. Other nutritional origins of anemia must be excluded by examining levels of folic acid, vitamin B12, zinc, copper, and vitamins A and E [22]. Eradication of *H. pylori *infection has been shown to improve responsiveness to iron supplementation and thus should be considered among the possible causes of postsurgical anemia [18].

### 6.2. Vitamin B12 and Folate Deficiency

Deficiencies of micronutrients folate and vitamin B12 causing anemia are less frequent after gastric bypass, because vitamin B12 storage depletion takes many years and high-dose oral vitamin B12 supplementation is very effective for prevention of this micronutrient deficiency [8, 9]. Similar to iron, the absorption of vitamin B12 is associated with hydrochloric acid in the stomach. This is the case because gastric acid is required for peptic digestion of the food-B12 complexes and because both hydrochloric acid and intrinsic factor are produced by gastric parietal cells. In addition to the mechanisms facilitated by hydrochloric acid, absorption of vitamin B12 in the terminal ileum requires the presence of intrinsic factor produced by the parietal cells of the stomach [8]. The primary reason for the occurrence of folate deficiency is a decreased dietary intake of folate. Folate is predominantly absorbed in the upper third of the small intestine, but absorption can occur along the entire length of the small intestine after surgical procedures. The incidence of folate deficiency following bariatric surgery has fallen dramatically in countries like USA where folic acid fortification of the food supply is practiced and is generally low in the face of widespread vitamin supplementation and nutrition support [9]. In addition, it has been suggested that the maintenance of normal folate status may be due to increased bacterial folate synthesis in the upper small bowel under achlorhydric conditions after gastric bypass surgery [9].

### 6.3. Copper Deficiency

The occurrence of copper deficiency has been reported in patients after RYGB. Copper is a trace metal that is absorbed in the stomach and proximal duodenum, both of which have disruption of their normal anatomy following GBP surgery. Copper is an important element for enzyme systems involved in hematopoiesis, catecholamine synthesis, and vascular and skeletal tissues as well as a key component in the structure and function of the nervous system. Following the formation of a gastrojejunostomy during RYGB, oral copper supplements may not be well absorbed by the jejunum. Animal studies suggest that the duodenum is the major site of copper absorption, but some absorption also occurs in the stomach and ileum [23]. Gastric pH has an important role in freeing copper bound to foodstuffs in natural organic complexes and ligands. Despite malabsorption being the likely mechanism underlying copper deficiency after RYGB, oral supplementation has been found to correct copper deficiency, suggesting that supersaturation of intestinal copper transport systems is a reliable way to maintain adequate serum levels [23]. Though the mechanism is poorly understood, it is known that intake of high doses of zinc can also induce copper deficiency [24].

Copper deficiency can manifest as hematologic disorders (anemia, neutropenia, and myelodysplasia) [25], as well as neurologic symptoms associated with demyelinating neuropathy which can mimic the findings in vitamin B12 deficiency [5, 8, 26]. Bone marrow findings of dysplasia with marked cytoplasmic vacuolization of erythroid and myeloid precursors, with occasional increased ring sideroblasts and hematogone hyperplasia, have been reported [10, 27]. Therefore, copper deficiency should be considered in the differential diagnosis of anemia, neutropenia, and bone marrow dysplasia in patients after bariatric surgery, especially for those presenting with neurologic deficits resembling subacute combined degeneration of the spinal cord [11]. 

Copper deficiency plays an important role in anemia in obesity because of its effects on iron absorption [28]. The mechanism of anemia in copper deficiency is possibly related to the role of copper-dependent enzymes, such as ceruloplasmin, hephaestin, and cytochrome-c oxidase in iron metabolism and transportation. Ceruloplasmin, which incorporates copper, is a ferroxidase that converts ferrous to ferric iron, facilitating its transport by transferrin. Hephaestin is a membrane-bound ceruloplasmin homolog that also incorporates copper. Deficiencies in hephaestin can lead to anemia due to hephaestin's role in iron egress from intestinal enterocytes [29]. Cytochrome-c oxidase, another copper-dependent enzyme, reduces ferric iron for its incorporation into the heme molecule. Therefore, copper deficiency interferes with iron transport and utilization at several key points and thus impairs heme synthesis, resulting in sideroblastic anemia [23]. The mechanism of neutropenia is unknown, although arrested granulocytic maturation has been postulated [10]. 

Although acquired copper deficiency is a rare cause of refractory anemia and leukopenia/neutropenia, it should be considered in the differential diagnosis of a patient with hematologic complications following bariatric surgery, particularly when there is a concomitant neurologic deficit. Measurement of serum copper and ceruloplasmin levels for confirmation would avoid a misdiagnosis of MDS or vitamin B12 deficiency and a possible delay in instituting appropriate therapy [8]. 

## 7. Bone Marrow Findings after Gastric Bypass Surgery Mimicking Myelodysplastic Syndrome

The obesity-related hematological complications are of clinical significance since anemia can result in severe comorbidities. Therefore, if anemia and single or multilineage cytopenias are present with no response to simple supplementation therapy after gastric bypass surgery, an early investigation by diagnostic bone marrow biopsy is warranted.

**Figure 2:**
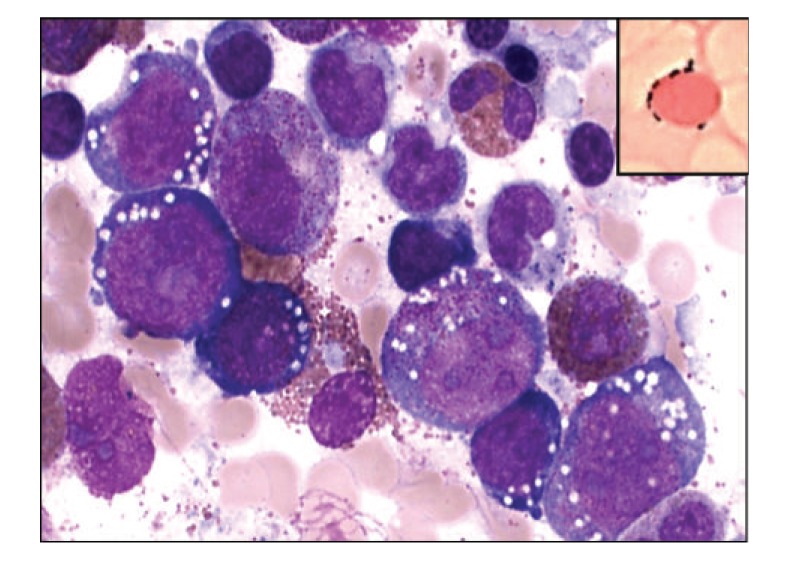
Bone marrow aspirate of a patient with history of gastric bypass surgery. The marrow smear shows vacuoles in the erythroid precursors, dyspoietic changes, and the presence of ringed sideroblasts (inset, Prussian Blue iron stain) (Wright Giemsa stain, original magnifications, ×500).

Although the bone marrow cells exhibit dysplastic features after gastric bypass surgery mimicking an underlying myelodysplastic syndrome (MDS) [25, 32], no convincing evidence of clonal abnormality in marrow stem cells is detected [30]. The marrow is hypercellular in most cases with characteristic findings of cytoplasmic vacuolization of both erythroid and myeloid precursor cells and a marked left shift in granulocytic precursors resulting in a decreased number of late and terminally differentiated myeloid cells, giving rise to the appearance of a myeloid arrest. Iron stores are characteristically increased with prominent ringed sideroblasts. These findings can be mistaken for a myelodysplastic syndrome [31]. Morphologic findings characteristic of MDS in the marrow include vacuolization of erythroid precursors, abnormal nuclear lobulation of both erythroid and myeloid precursors, nuclear/cytoplasmic dyssynchrony of erythroid precursors, and dysmegakaryopoiesis with abnormalities of nuclear lobulation and size [25, 27]. Abnormal ringed sideroblasts sometimes can be observed (Figure 2).

There is evidence that copper deficiency is commonly seen in patients with apparent MDS following gastric reduction surgery and in whom administration of copper results in the reversal of the dysplastic changes [11, 25, 32]. The decreased copper level in MDS might be a cause of iron overload and therefore an initiating factor of the dysplastic process [28, 33]. The mechanism of cytopenia(s) remains unknown, but recent studies suggest that copper deficiency results in the inhibition of differentiation and self-renewal of CD34+ hematopoietic progenitor cells [34]. 

## 8. Obesity Mediated Inflammatory Anemia after Gastric Bypass Surgery

Inflammation arising from various etiologies, including infection, autoimmune disorders, chronic diseases, and aging, can promote anemia [2, 8]. Obesity is also characterized by chronic inflammation, which is aggravated by rapid weight loss during the early postoperative period following RYGB. It was postulated that the associated elevation of a variety of proinflammatory cytokines increases the likelihood of anemia of inflammation and can also induce myelodysplastic-like features in the marrow that persist in the longterm after RYGB [22]. Hepcidin is upregulated by interleukin-6, and these factors act with other inflammatory cytokines and proteins, such as TNF-*α*, von Willebrand factor, C-reactive protein, and fibrinogen, which are all elevated in the early post-RYGB period [21]. These interactions disrupt normal hematopoiesis and lead to anemia and other cytopenias. Anemia relating to this inflammatory state is also associated with alterations in erythrocyte physiology, including decreased lifespan and impaired biological responsiveness to erythropoietin [35]. In the long-term period after surgery, this may be further complicated by underlying copper and other micronutrient deficiencies [22].

**Figure 3:**
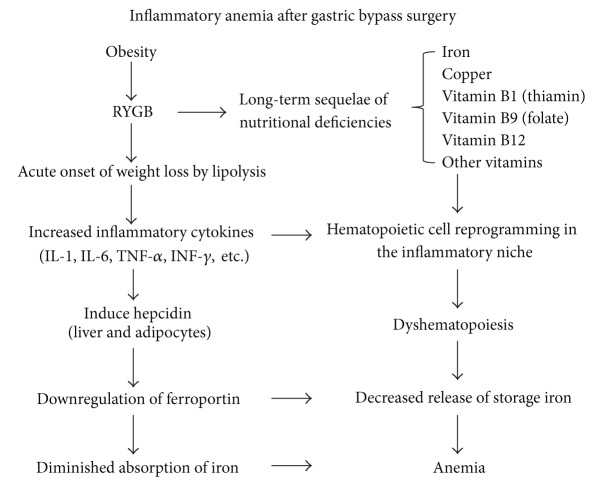
Proposed mechanistic link between obesity and inflammatory anemia after gastric bypass surgery.

A chronic low-grade systemic inflammatory response is a defining feature of obesity [4]. This smoldering inflammation in tissues, whether in adipose tissue or bone marrow, is primarily mediated by the innate immune system [36, 37]. Currently, adipose tissue is considered an endocrine and immune-modulating organ that is able to mediate biological effects on metabolism and inflammation, contributing to the maintenance of energy homeostasis and the pathogenesis of obesity-related metabolic and inflammatory complications [13]. Obesity causes chronic inflammation, which is associated with the expression and release of proinflammatory cytokines [4]. Proinflammatory cytokines such as tumor necrosis factor-alpha (TNF*α*) and interferon-gamma- (IFN-*γ*) have been shown to negatively affect erythropoiesis and iron homeostasis both *in vitro *and *in vivo *[38, 39]. While the mechanisms have not been fully elucidated, it is clear that obesity-related chronic inflammatory stimuli to the bone marrow microenvironment provide a unique niche for interaction between mesenchymal stromal/stem cells (BMSCs) and hematopoietic stem cells (HSCs) [40]. The plasticity of bone marrow-derived stem cells in the inflammatory bone marrow niche and immune dysregulation may explain some of the morphological changes seen after gastric bypass surgery [22]. Proinflammatory cytokines may result in the release of hepcidin from the liver or adipose tissue [15]. Hepcidin is an important regulator of iron homeostasis, inhibiting iron absorption at the enterocyte and sequestering iron at the macrophage, which could lead to increased iron stores in conjunction with hypoferremia (the anemia of inflammation) [14, 21]. The potential role of hepcidin in the development of iron deficiency in the obese is supported by the discovery of elevated hepcidin levels in tissue from patients with severe obesity [14] and the positive correlation between adipocyte hepcidin expression and body fat index [14, 21] (Figure 3). Hepcidin appears to block iron uptake in the duodenum and iron release from macrophages, thereby decreasing delivery of iron to erythroid precursors [21, 41]. Even though the hepcidin-inflammation connection provides a succinct and logical biological framework to explain the association of iron deficiency with obesity in the postsurgery setting, additional research is required to clarify the biological mechanisms underlying this relationship. 

The state of systemic inflammation associated with obesity is exacerbated immediately after RYGB due to increased lipolysis and fat oxidation during rapid weight loss [2, 12]. Disruption of neuronal, hormonal, and inflammatory pathways can induce abnormal cytokine production, increased levels of acute-phase reactants, and activation of inflammatory signaling pathways. The cross talk between metabolic disorders, chronic inflammation, and immune dysregulation on the one hand and long-term sequelae of nutritional deficiency on the other may lead to a complex suppression of normal hematopoiesis that often does not respond to the simple replacement of deficient micronutrients [8, 42]. Thus, following RYGB, the early exacerbation of systemic inflammation can trigger persistent anemia of chronic disease despite nutritional supplementations during long-term followup. The chronic inflammation, with specific reference to bone marrow macrophages, has detrimental effects on hematopoietic stem cells [8, 22, 43]. The temporal and spatial relationships of the inflammatory response target marrow cells that are critical in the normal function of the stromal environment in the bone marrow [43]. Critical questions remain concerning the underlying molecular mechanisms and this unique type of inflammatory anemia may require a distinct therapeutic approach. 

## 9. Conclusion

In summary, bariatric surgery is successful in inducing weight loss in morbidly obese individuals, but often is complicated by resultant hematological disorders. The evaluation of anemia and single or multilineage cytopenias after gastric bypass surgery must take into account the unique features of the RYGB clinical setting. Attention to the time of onset of the cytopenia(s) is important, because inflammation, drugs, and infections are more likely to occur in the first few months after surgery, either as the direct agent of marrow suppression or as the trigger for immune cytopenia(s) [37, 44]. Malnutrition including iron, copper, and B12 deficiencies should always be investigated as a potential precipitating or aggravating cause of cytopenia(s). Drug-related anemia and cytopenia(s) due to a variety of mechanisms, including perturbation of T-cell subsets leading to autoimmune cytopenia, should also be considered [22]. Early investigation of the etiology of persistent cytopenia(s) by diagnostic bone marrow biopsy is warranted, because the cytopenia conditions usually have a better prognosis if early interventions are undertaken [7].
